# Monitoring Gut Epithelium Serotonin and Melatonin Overflow Provides Spatial Mapping of Inflammation

**DOI:** 10.1002/cbic.202200334

**Published:** 2022-12-02

**Authors:** Fernando Perez, Nikki Kotecha, Brigitte Lavoie, Gary M. Mawe, Bhavik Anil Patel

**Affiliations:** ^1^ Centre for Stress and Age-Related Disease School of Applied Sciences University of Brighton Brighton BN2 4GJ UK; ^2^ Department of Neurological Sciences The University of Vermont Burlington VT-05405 USA

**Keywords:** EC cells, electrode arrays, inflammation, melatonin, serotonin

## Abstract

Electrochemical arrays were used to measure the overflow of serotonin (5‐HT) and melatonin (MEL) from the entire colon of healthy mice and mice with chemical‐induced inflammatory bowel disease (IBD), to understand the interplay between inflammation and colonic function. We show that 5‐HT overflow is increased, whilst MEL levels are reduced, in inflamed tissues. The levels of MEL are increased at the interface between healthy and inflamed regions within the colon and may limit the spread of inflammation. Understanding the interplay between inflammation and mucosal epithelial signalling can provide key insight into colonic function and aid the development of effective therapeutic strategies to treat gastrointestinal diseases.

## Graphical abstract

**Serotonin and melatonin** play critical roles in regulating gut motility, but also have a key influence on the inflammatory status of the gut. The overflow of serotonin and melatonin provided spatial mapping of inflamed regions within the colon. This finding provides new insight into the tracking and therapeutic management of inflammatory bowel disorders.

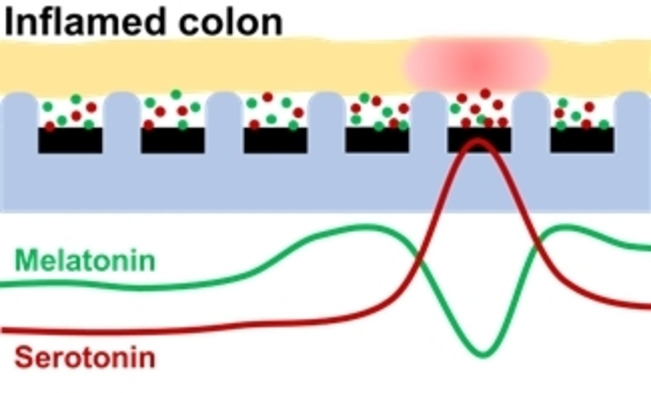

## Introduction

Inflammatory bowel disease (IBD) is a chronic intestinal inflammatory condition, which is comprised of two main types, ulcerative colitis (UC) and Crohn's disease (CD). When the intestinal tract is inflamed, there is breakdown of intestinal barrier function, abnormal secretion, changes in the patterns of motility and visceral sensation, all of which contribute to symptoms such as diarrhoea and abdominal pain. UC causes inflammation and ulcers in the mucosal epithelial lining of a continuous region of the colon. In CD, full thickness lesions are interspersed with regions of normal healthy gut. Within the mucosal epithelium resides enterochromaffin (EC) cells, which release serotonin (5‐hydroxytryptamine; 5‐HT) and melatonin (MEL) following either chemical or mechanical stimulation.^[1]^ EC cells serve as epithelial transducers between the lumen and the underlying neuronal and glial network of the enteric nervous system.^[2]^ EC cells can function as a key regulator of propulsive motility patterns.^[3]^ Studies of animal models and human subjects have demonstrated inflammation‐related alterations in 5‐HT and recently MEL. Alongside key roles in motility, these molecules also have a balanced relationship with regards to the regulation of inflammation. Elevated 5‐HT can induce inflammation and exacerbate colitis.^[4]^ By contrast MEL is well known to act as an antioxidant and reduce markers of inflammation.^[5]^ Given the significant interplay between epithelial signalling molecules and inflammation, a detailed understanding of how mucosal signalling is altered spatially over inflamed regions is crucial to dissect the locations of healthy and diseased tissues. Electrochemical techniques are recognized as valuable tools to monitor epithelial signalling molecules, as they allow real time monitoring of 5‐HT and MEL overflow with high temporal and spatial resolution. Electrochemical arrays with multiple potential amperometry provides for the means to monitor both 5‐HT and MEL over large regions of tissues and thus provide the ability to conduct whole mapping of the colon.

In this study, we investigated the overflow of 5‐HT and MEL in wildtype (WT), dextran sodium sulfate (DSS)‐induced (model of UC) and trinitrobenzene sulfonic acid (TNBS)‐induced (model of CD) murine colons to understand the spatial variations in the signalling of these molecules in the regions of inflammation. In the trinitrobenzene sulfonic acid (TNBS) colitis model, there is a self‐limited inflammatory response following a single enema administration in ethanol. TNBS colitis involves primarily an adaptive immune response that is largely Th1 mediated,^[6]^ although a Th2 component has been reported in BALB/c mice.^[7]^ In the other model, dextran sodium sulfate (DSS) is added to the drinking water. DSS colitis involves an innate immune response since DSS colitis occurs in immunodeficient mice.^[8]^ While these models do not perfectly reflect the aetiologies of the two major forms of inflammatory bowel disease, TNBS and DSS colitis mimic several prominent clinical and morphological features of CD^[9]^ and UC,^[10]^ respectively.

## Results and Discussion

### Characterisation of the 3D printed electrochemical array for robust measurement of 5‐HT and MEL

**Figure 1:**
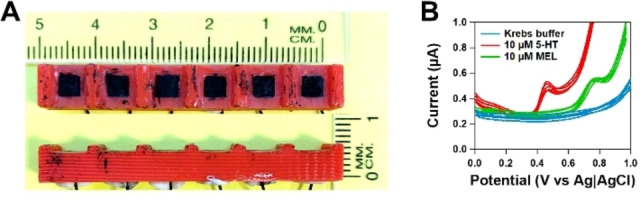
Electrochemical array for monitoring 5‐HT and MEL from an entire mouse colon. (A) Photograph of the electrode array, which contains six 4×4 mm MWCNT composite electrodes. The electrode array contains 1 mm raised bar between the working electrodes to allow fixed distance between the electrode and tissue at all regions of the colon. (B) Differential pulse voltammograms of 10 μM 5‐HT and MEL and the blank Krebs buffer, showing the oxidation peak potentials for both signalling molecules.

A 3D printed housing for six MWCNT epoxy composite electrodes was used (Figure 1A). The device contained 1 mm raised bars between the electrodes to maintain a constant distance between the colon and electrodes, aid the flow of physiological media, and minimize electrode crosstalk.^[11]^ This approach towards making large electrochemical arrays was shown to be reproducible when comparing the electrochemical response to a standard redox probe between electrodes within an array or between multiple electrochemical arrays (Figure S1).

5‐HT was oxidised at +480 mV and MEL was oxidised at +780 mV (Figure 1B) and therefore for multiple step amperometry measurements, the electrodes were held at +300 mV, +650 and +800 mV versus an Ag|AgCl reference electrode.

Calibration responses were conducted for 5‐HT and MEL (2–10 μM), where there was good precision over the six working electrodes (Figure S2 A&B). For the detection of MEL, a linear relationship between current and concentration was observed at +800 mV, but no current change was observed when run at +650 mV, indicating no interference from 5‐HT. For 5‐HT the sensitivity was 30 nA μM^−1^ and the limit of detection (LOD) was 0.2 μM. For MEL the sensitivity was 31 nA μM^−1^ and the limit of detection (LOD) was 0.27 μM (Figure S2 C&D). Fouling studies were conducted to explore the stability of the electrochemical array for monitoring 5‐HT and MEL (Figure S3). For measurements, the reduction peak current and potential of redox probe ruthenium (III) hexaamine was monitored using differential pulse voltammetry following exposure to 10 μM 5‐HT or MEL every 30 s using amperometry (Figure S3A–D). Over the duration of 150 s, there was a significant decrease in the current response of ruthenium (III) hexaamine following measurements in 10 μM 5‐HT (p<0.001, n=6, Figure S3E). When compared to the initial response, there was a reduction in the ruthenium (III) hexaamine current at 60 s (p<0.01), 90 s (p<0.01), 120 s (p<0.05) and 150 s (p<0.01). The cathodic peak potential (EpC) for ruthenium (III) hexaamine was also reduced at 120 s (p<0.001) and 150 s (p<0.01, n=6; Figure S3 F) when compared to the initial response, when exposed to 5‐HT. These findings indicate that the response of the MWCNT composite electrode to 5‐HT is stable for 30 s, and then is prone to fouling from oxidative by‐products of the 5‐HT oxidation, which other have also shown.^[12]^ However, for the detection of MEL, there was no significant difference in the current and EpC of ruthenium (III) hexaamine over the 150 s (Figure S3 E&F). These findings highlight that the MWCNT composite electrode array has the sensitivity and stability to accurately measure 5‐HT and MEL overflow from the entire mouse colon.

### Understanding the relationship between inflammation, 5‐HT and MEL over the entire colon

**Figure 2:**
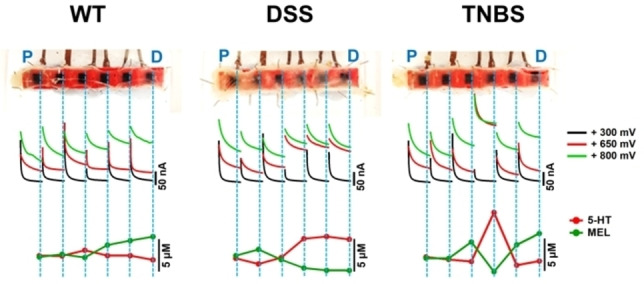
Measurement of 5‐HT and MEL from WT, DSS‐inflamed and TNBS inflamed colon. The entire colon was pinned over the electrode device where P indicates the proximal region of the colon and D indicates the distal region of the colon. Amperometric measurements were conducted at each electrode for 30 s at three different voltages (+300, +650 and +800 mV). The difference in the current at +650 and +300 mV provided the 5‐HT response. The difference in the current at +850 and +650 mV provided the MEL response. The average current in the last 5 seconds of the amperometric trace at each electrode was converted to the 5‐HT and MEL concentration using calibration response.

We have previously shown using HPLC and DPV measurements that only 5‐HT and MEL are the only released molecules that can be detected from colonic tissue.[^1c^, ^13^] Amperometric measurements were obtained at three different voltages to monitor the overflow of 5‐HT and MEL on WT, DSS‐inflamed and TNBS‐inflamed over the entire mouse colons (Figure 2). The current difference between +300 mV and +650 mV represents the response of 5‐HT and the current difference between +650 mV and +800 mV represents the response of MEL. Clear differences were detected in the overflow of 5‐HT and MEL over different regions of the bowel and in the presence of inflammation.

**Figure 3:**
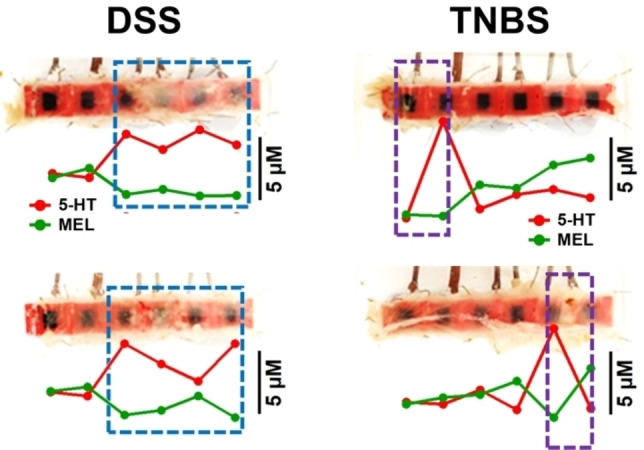
The link between inflammation of the colon and overflow of 5‐HT and MEL. The boxes highlight regions within DSS‐inflamed and TNBS‐inflamed colon, which show visual and macroscopic signs of inflammation. The associated profile of 5‐HT and MEL for these individual colons is shown, where at the region of inflammation, increases in 5‐HT and decreases in MEL were observed.

The associations between 5‐HT and MEL with inflammation are highlighted in Figure 3. Following DSS and TNBS treatment, only specific areas of the entire colon were inflamed. Inflammatory regions were observed through visual inspection, by observing changes in the transparency and colour change in the tissue. In areas where clear visual signs of inflammation occurred there was an increase in 5‐HT overflow and a decrease in MEL overflow. This indicates there are clear changes in the signalling of 5‐HT and MEL in inflamed tissue regions. These changes are consistent with previous studies of 5‐HT in DSS‐ and TNBS‐inflamed colons, but present new information for MEL. The spatial mapping also provides interesting observation on these signalling molecules at the boundaries between inflamed zones, where a greater concentration of MEL is present, potentially showcasing an anti‐inflammatory role for MEL to reduce and contain the degree of inflammatory damage.

**Figure 4:**
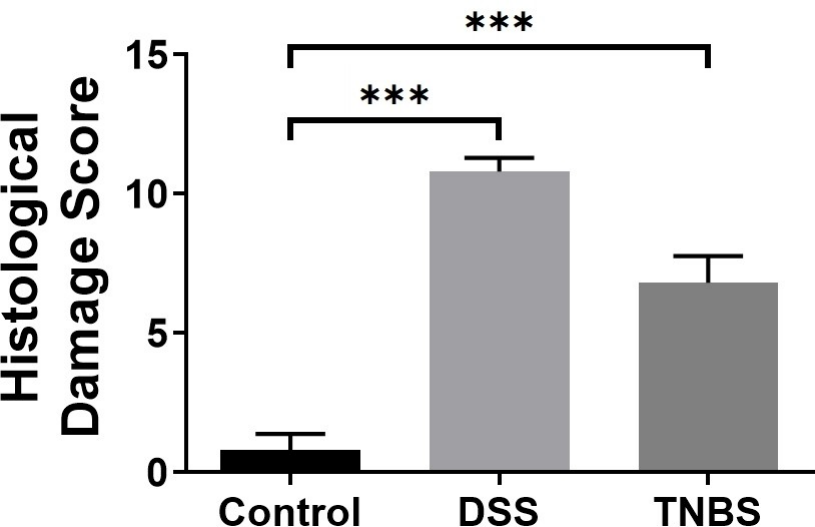
Histological damage score recorded from 1 cm specific regions of the colon of WT, DSS‐treated and TNBS‐treated animals that visually different due to changes in transparency and colour of the colon. Data shown as mean±s.d., n=5, where ***P<0.001.

The changes in 5‐HT and MEL are linked to the type of inflammation observed within these two model systems. DSS‐inflamed model is comparable to UC with large continuous patch of inflammation on the mucosal epithelium while TNBS‐inflamed model shares more similarities with CD, in which patches of inflamed regions are interspersed with healthy areas. The specific regions highlighted in the boxes in Figure 3 were used for histological assessment to verify the presence of inflammation. The damage was shown to be significantly greater in DSS and TNBS‐inflamed colons (<0.001, n=5, Figure 4) when compared to WT colons.

### Individual variations in the patterns of mucosal signalling are correlated with inflammation of the mucosal epithelium

**Figure 5:**
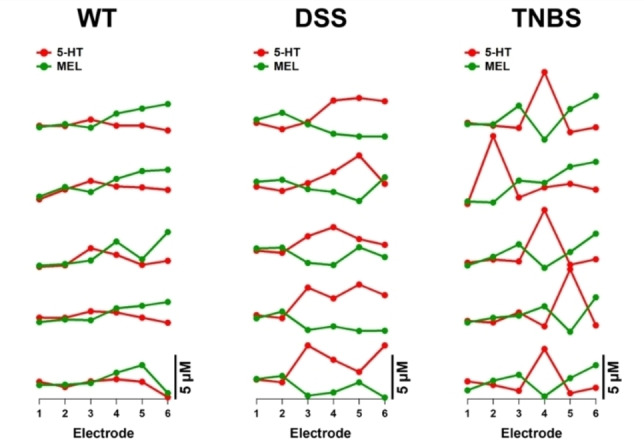
Individual responses of whole colon mapping from WT, DSS‐inflamed and TNBS‐inflamed colon. Red traces represent 5‐HT and green traces represent MEL. Electrode 1 is at the proximal most region of the colon and electrode 6 is at the distal most region of the colon.

Observations from multiple colons reveal that spatial mapping of the changes in 5‐HT and MEL is specific to inflammation as highlighted in Figure 5, especially in TNBS inflamed animals, where inflamed regions present at different locations within the colon. Electrodes 1–6 represent the proximal to distal regions of the colon. For 5‐HT, similar responses were observed when comparing individual WT vs. DSS colons (Figure 5). Overall, responses demonstrate a significant increase in 5‐HT levels in DSS‐inflamed colons at electrode 4 (p<0.05), 5 and 6 (p<0.01, n=5, Figure S4 A). A similar comparison between WT and TNBS colons was not performed given the heterogeneous distribution of inflamed regions observed between the colons. For TNBS‐inflamed colons spikes in 5‐HT levels were observed over a single electrode but at varying positions from electrode 1–6 (Figure 5). It is clear from our findings that when inflammation is present, 5‐HT levels are elevated, as has been observed by other studies.[^13b^, ^14^] These findings may be due to increased release, greater expression of EC cell and reduction/loss in serotonin transporter (SERT) function.

**Figure 6:**
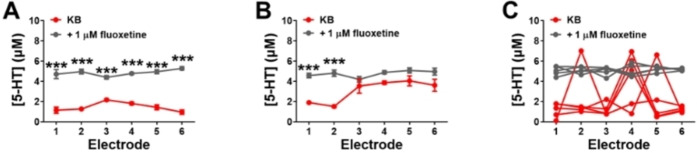
Effect of 1 μM fluoxetine on 5‐HT overflow. Response on (A) WT, (B) DSS‐inflamed and (C) TNBS‐inflamed colons. Electrode 1 is at the proximal most region of the colon and electrode 6 is at the distal most region of the colon. Data shown as mean±s.d., n=6, where *p<0.05, **p<0.01 and ***p<0.001.

To explore this further we investigated how the SERT blocker 1 μM fluoxetine would influence the observed levels of 5‐HT (Figure 6). In WT colons, a significant increase in 5‐HT overflow was observed on electrodes 1–6 in the presence of 1 μM fluoxetine (p<0.001, n=5, Figure 6A). For DSS‐inflamed colons there was significant increase in 5‐HT overflow on electrodes 1 and 2 in the presence of 1 μM fluoxetine (p<0.001, n=5, Figure 6B). No significant difference in the current was observed on electrodes 3–6 in the presence of 1 μM fluoxetine in DSS‐inflamed colons. For TNBS‐inflamed colons, responses from each colon are shown in the presence and absence of 1 μM fluoxetine (Figure 6C). When elevated levels of 5‐HT were observed at specific electrodes from individual colons, they were not different to the currents observed in the presence of 1 μM fluoxetine. These findings indicate that in the presence of inflammation, the SERT function is decreased, which has been observed in other studies that have used DSS‐ and TNBS‐inflamed models.[^4a^, ^14b^, ^14c^, ^15^] The elevated level of 5‐HT is also most likely exacerbating the inflammatory status. This was highlighted by a seminal study in which DSS‐inflamed tryptophan hydroxylase 1 knockout mice had significantly reduced mucosal 5‐HT content, down‐regulation in the severity of colitis and a reduction in pro‐inflammatory cytokine, TNF‐α, IL‐1β, and IL‐6 production when compared to WT mice.^[4b]^

MEL levels shown in Figure 5 were very similar when comparing individual WT and DSS‐inflamed colons. When considering the overall response, MEL levels were significantly decreased in electrodes 4 (p<0.001), 5 (p<0.01) and 6 (p<0.001, n=5) in DSS‐inflamed colons when compared to WT colons (Figure S4B). In TNBS‐inflamed colons, MEL levels in the vicinity of inflammation were significantly decreased, but elevated MEL levels were observed at the edges of inflammation (Figure 5). These key findings highlight that production of antioxidant MEL is reduced by the onset of inflammation, most likely due to the loss of the key synthesis enzymes arylalkylamine‐*N*‐acetyltransferase (AANAT) and hydroxyindole‐*O*‐methyl‐transferase (HIOMT). A new observation from this study is the elevation of MEL on the edges of inflammation in both DSS and TNBS‐inflamed colons, which is critical given that MEL can reduce markers of inflammation and thus showcases the change in signalling to help reduce the extent of inflammation within the colon.

Our previous studies in various model systems have highlighted that in all WT models, MEL is present at higher levels than 5‐HT.[^1c^, ^13a^, ^13b^] This is more evident in the distal colon, a region more susceptible to oxidative damage and likely serves to ensure the health status of the colon. When the MEL : 5‐HT is below 1, there is a pro‐inflammatory state and when the level is above 1, this supports a healthy status. In Figure S4C, the MEL to 5‐HT ratio is presented for WT and DSS‐inflamed colons. When compared to WT, there is a significant reduction in the MEL : 5‐HT in DSS‐inflamed animals at electrode 4 (p<0.05), 5 (p<0.001) and 6 (p<0.001). This indicates a very strong emphasis towards pro‐inflammatory status and thus significant exacerbation of the inflammatory status is likely. Whist in TNBS‐inflamed colons, at the individual regions were 5‐HT levels spike and MEL levels are reduced, the MEL : 5‐HT ratio is below 1 and is limited to one electrode region in each individual colon (data not shown). However, in all other areas it closely matches that of the WT colon. This underscores the very region‐specific inflammation that occurs in TNBS‐inflamed colons and those who suffer CD.

## Conclusion

In summary, multiple potential amperometry with a MWCNT composite electrode array provides the ability to investigate the spatial overflow of 5‐HT and MEL over the entire colon. Using two different inflammation models, our results show that 5‐HT is elevated in zones that are inflamed, whilst MEL is reduced in these regions. These alterations in mucosal signalling could drastically influence the inflammatory status of the tissue, as 5‐HT will exacerbate the extent of inflammation. One the edges of inflamed regions, MEL levels are elevated. This provided a degree of protection to reduce the spread and extent of the inflammation within the colon. These findings highlight that mucosal signalling markers provide key insight into locality of inflammation and importantly these localized changes within the colon will have significant impact of the motility within the colon. Our findings clearly demonstrate the balance of 5‐HT and MEL are critical to the inflammatory status and colonic function. Therapeutic strategies to influence the levels of 5‐HT and MEL may thus have a profound impact on regulating the inflammatory status of the bowel.

## Experimental Section

**Fabrication of the electrode array**: The 3D printed house was designed using SolidWorks (Dassault Systèmes) and printed using raise 3D pro (Raise 3D Technologies, Inc., Irvine, CA) using poly‐lactic acid (PLA). The device was 50 mm long and 7 mm wide which can span the entire length of the murine colon. Six working electrodes were of the dimensions 4×4×2 mm, with a hole in the centre of the electrode slot to connect with a silver wire to establish ohmic connection. At the top of the housing, 1 mm raised bars were present in‐between the electrodes to ensure consistent distance between the electrode and the tissue. The electrodes within the device were made using 15 % w/w of MWCNT to 85 % of epoxy resin/hardener mix (where epoxy resin to hardener ratio was 3 : 1, Robnor Resins Ltd. Swindon, Wilts, UK), which we have previously shown to have good conductivity. Prior to use, the electrodes were mechanically polished using fine sandpaper, followed by alumina slurry. The completed electrode array was embedded in a Sylgard®‐(Dow Corning, UK) lined Teflon recording chamber for characterisation and biological measurements.

**Electrochemical characterisation of the electrode array**: All measurements were conducted using a 3‐electrode system using a Ag|AgCl reference electrode and Pt wire as the counter electrode. Recordings were obtained using CH1030B multichannel potentiostat (CH Instruments, Austin, TX). To assess electrodes within a single array and between arrays, measurements were conducted in 1 mM ruthenium (III) hexaamine in 1 M KCl (Sigma Aldrich, UK) using cyclic voltammetry at 100 mVs‐1. Calibrations were conducted in the range of 2 to 10 μM 5‐HT and MEL in a modified Krebs buffer (pH 7.4; 117 mM NaCl, 4.7 mM KCl, 2.5 mM CaCl2, 1.2 mM MgCl2 and 1.2 mM NaH2PO4). For the 5‐HT calibration, measurements were conducted at +650 mV and +300 mV. For the MEL calibration, measurements were conducted at +800 and +650 mV vs Ag|AgCl reference electrode. For fouling studies, measurements were conducted using solutions of 1 mM ruthenium (III) hexaamine either containing 5‐HT or MEL in Krebs buffer. Differential pulse voltammograms of 1 mM ruthenium (III) hexaamine were obtained between the voltages of +200 to −500 mV in between amperometric runs in either 10 μM 5‐HT (electrodes held at +650 mV) or MEL (electrodes held at +800 mV) for a duration of 30 s. This protocol was repeated 5 times to record the response of 1 mM ruthenium (III) hexaamine after exposure to either 5‐HT and MEL for 150 s.

**Animal models**: Twenty‐five male 8‐week‐old CD1 mice (Charles River) were used in this study. DSS colitis was induced in mice by administering DSS (wt/vol in water; 3–4 %; molecular weight: 36,000–50,000; MP Biomedicals, Solon, OH) in drinking water for 5 days. Mice were return to tap water after the induction. TNBS colitis was induced by a single colonic enema of TNBS (7.5 mg/ml in 50 % ethanol; 100 uL) delivered under anaesthesia. Mice were euthanized by isoflurane overdose and exsanguination one week after induction.

**Microscopic scoring of inflammation**: After electrochemical measurement, the portion of the distal colon (1 cm) in which evaluated 5‐HT and reduced MEL were observed was collected and fixed in 4 % paraformaldehyde. The colons were paraffin embedded, sectioned and H&E stained for histological assessment of colitis. Histological damage scores were assigned using a scoring rubric modified from one commonly used in human which accounts for common features of IBD, namely altered crypt architecture, invasion of lymphoid cells into the lamina propria and areas of eroded epithelial layer. The slides were evaluated by an observer blinded to knowledge of treatment group.

**Measurements from the entire colon**: The colon was obtained by removing the cecum and rectum and was between 6–7 cm in WT but contracted to 5 cm in DSS‐inflamed and TNBS‐inflamed animals. The colon opened along its mesenteric border and placed with the mucosal layer facing the electrodes. The tissue was stretched around the electrode array and pinned to the adjacent Sylgard. The Teflon recording chamber was perfused with warm (37 °C) Krebs buffer solution (pH 7.4; 117 mM NaCl, 4.7 mM KCl, 2.5 mM CaCl2, 1.2 mM MgCl2, 1.2 mM NaH2PO4, 25 mM NaHCO3 and 11 mM glucose) at a flow rate of 2 mL/min. Tissues were perfused for 30 minutes prior to commencing a series of measurements. For measurements of 5‐HT and MEL overflow, amperometric recordings were conducted on all electrodes for a duration of 30 s at +300, +650 and +850 mV vs. Ag|AgCl reference electrode. Measurements were conducted on WT, DSS‐inflamed and TNBS‐inflamed colons. To explore the function of SERT, tissues were perfused with 1 μM fluoxetine in Krebs buffer for 30 minutes prior to conducting measurements.

**Data analysis and statistics**: For amperometric recordings, the average current at the different potentials applied was obtained between 25–30 s at each electrode. For determination of 5‐HT, the current different between amperometric recordings at +300 and +650 mV was obtained. For the determination of MEL, the current different between amperometric recordings at +650 and +800 mV was obtained. The current was then converted to the concentration of 5‐HT and MEL using the calibration responses obtained. All statistics were performed in GraphPad Prism 9 (GraphPad Software Inc., San Diego, CA) with unpaired t‐test, one‐way or two‐way ANOVA depending on the type of experiment. Statistical significance was designated at p <0.05 and all data are presented as mean of median±SEM.

All procedures involving mice were approved by the University of Vermont Institutional Animal Care and Use Committee.

## Conflict of interest

The authors declare no conflict of interest.

1

## Supporting information

As a service to our authors and readers, this journal provides supporting information supplied by the authors. Such materials are peer reviewed and may be re‐organized for online delivery, but are not copy‐edited or typeset. Technical support issues arising from supporting information (other than missing files) should be addressed to the authors.

Supporting Information

## Data Availability

The data that support the findings of this study are available from the corresponding author upon reasonable request.
