# Supplementary material for: Monitoring Gut Epithelium Serotonin and Melatonin Overflow Provides Spatial Mapping of Inflammation
Source: Chembiochem. 2022 Dec 2;24(2):e202200334. doi: 10.1002/cbic.202200334 (PMC9909162; doi:10.1002/cbic.202200334)
Supplement: Supplementary file 1 — Supporting Information [file CBIC-24-0-s001.pdf]

ChemBioChem

Supporting Information

Monitoring Gut Epithelium Serotonin and Melatonin Overflow Provides Spatial Mapping of Inflammation

Fernando Perez, Nikki Kotecha, Brigitte Lavoie, Gary M. Mawe, and Bhavik Anil Patel*

1. *Figure S1* – Variability between electrodes within a device and between devices
2. *Figure S2* – Calibration responses for 5-HT and MEL
3. *Figure S3* – Stability of electrodes for repeated measurements of 5-HT and MEL
4. *Figure S4* – The Effect of DSS induced inflammation on mucosal epithelium 5-HT and MEL overflow

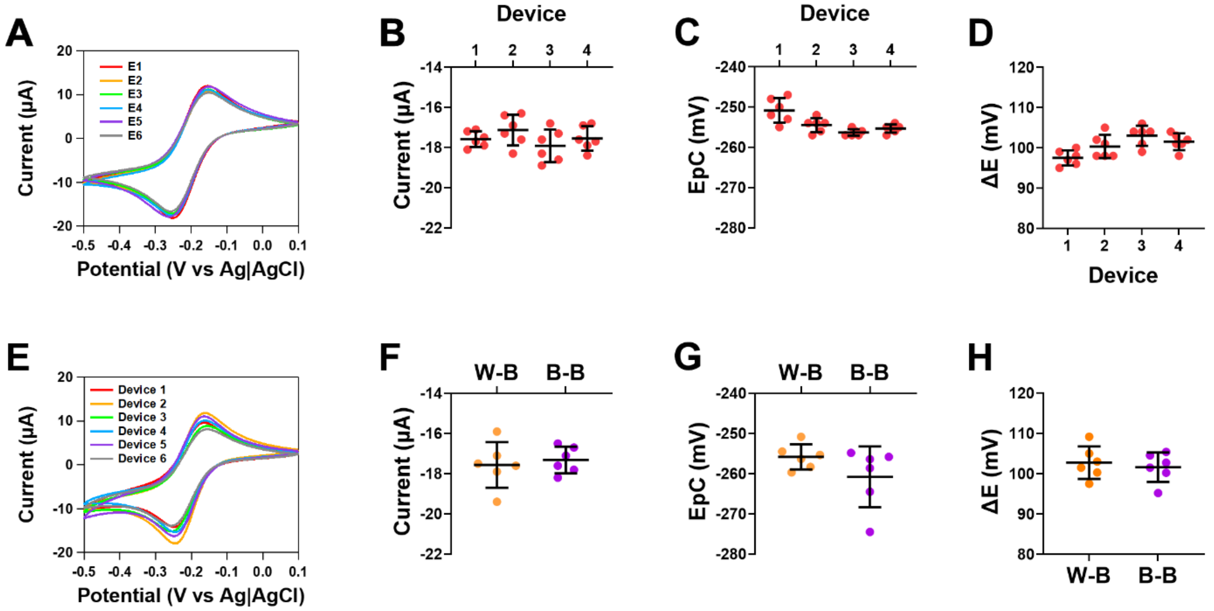

Figure S1. Variability between electrodes within a device and between devices. (A) cyclic voltammograms of 1 mM ruthenium (III) hexaamine on all electrodes within a single device. (B) Cathodic peak current, (C) cathodic peak potential (EpC) and (D) difference between the cathodic and anodic peak potential (ΔE) shown for all electrodes over 4 different devices. (E) the average cyclic voltammogram of 1 mM ruthenium (III) hexaamine of a single electrode array, where comparisons of multiple arrays are shown. (F) Cathodic peak current, (C) EpC and (D) ΔE showing the variation within a batch (W-B) and between a batch (B-B) of electrode arrays. Data shown as mean ± St.Dev., n=6 for electrodes and devices

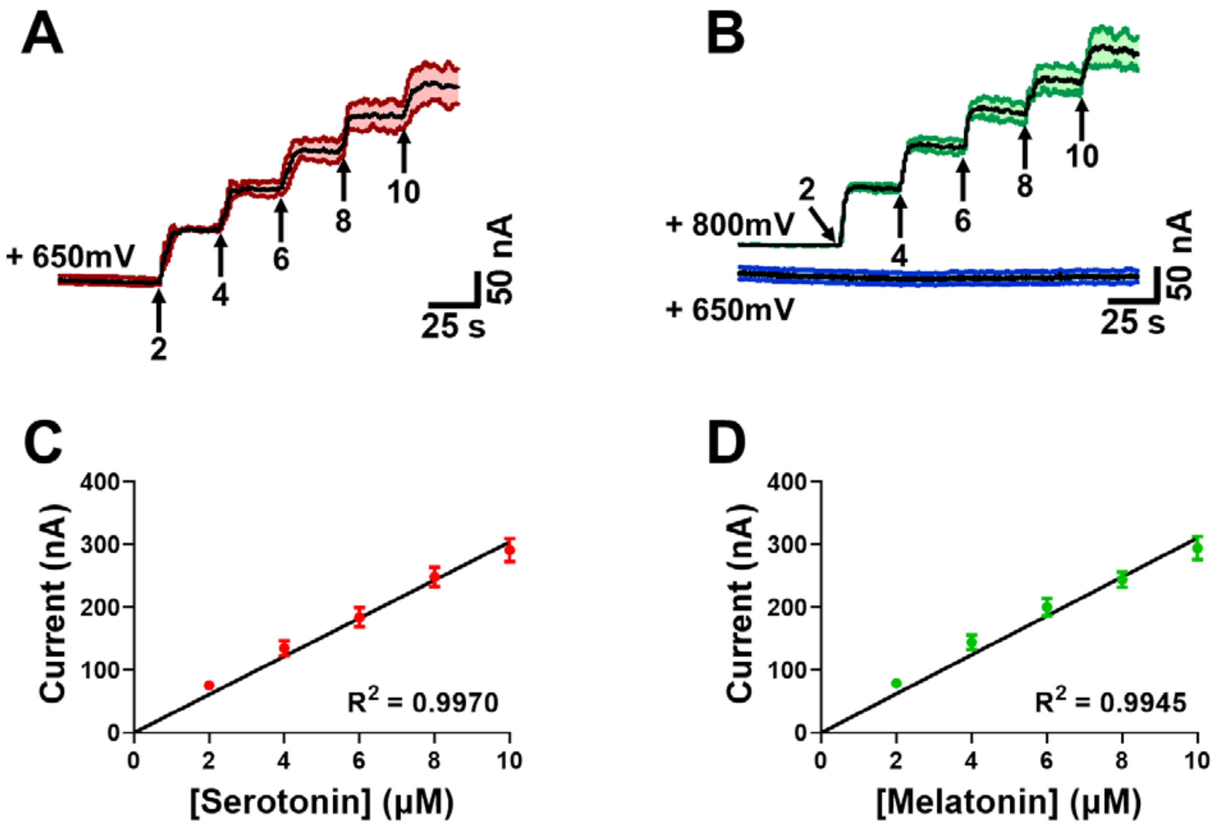

Figure S2. Calibration responses for 5-HT and MEL. (A) Amperometric responses at +650 mV for the measurement of 5-HT. (B) Amperometric responses at +650 mV and + 800 mV for the measurement of MEL. Values highlight concentration in µM. The shaded response highlights the range of responses obtained in varying devices. Calibration responses for (C) 5-HT and (D) MEL. Data shown as mean ± St.Dev, n=6.

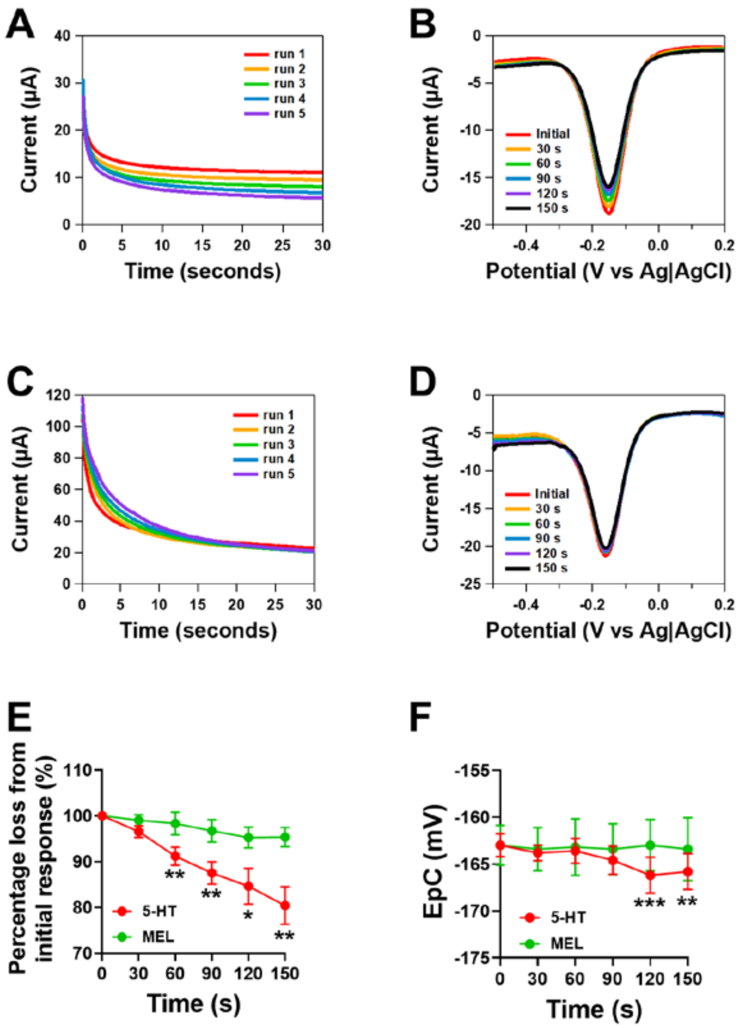

Figure S3. Stability of electrodes for repeated measurements of 5-HT and MEL. (A) amperometric recordings of 10 µM 5-HT at +650 mV Vs Ag|AgCl reference electrode made in-between (B) differential pulse voltammograms of 1 mM ruthenium (III) hexaamine in modified Krebs buffer. (C) amperometric recordings of 10 µM MEL at +800 mV Vs Ag|AgCl reference electrode made in-between (D) differential pulse voltammograms of 1 mM ruthenium (III) hexaamine in modified Krebs buffer. (E) Percentage loss from the initial response of ruthenium (III) hexaamine following exposure to 5-HT and MEL. (F) Changes in EpC of ruthenium (III) hexaamine following exposure to 5-HT and MEL. Data shown as mean ± St.Dev., n=6, where *$p<0.05$, **$p<0.01$ and ***$p<0.001$.

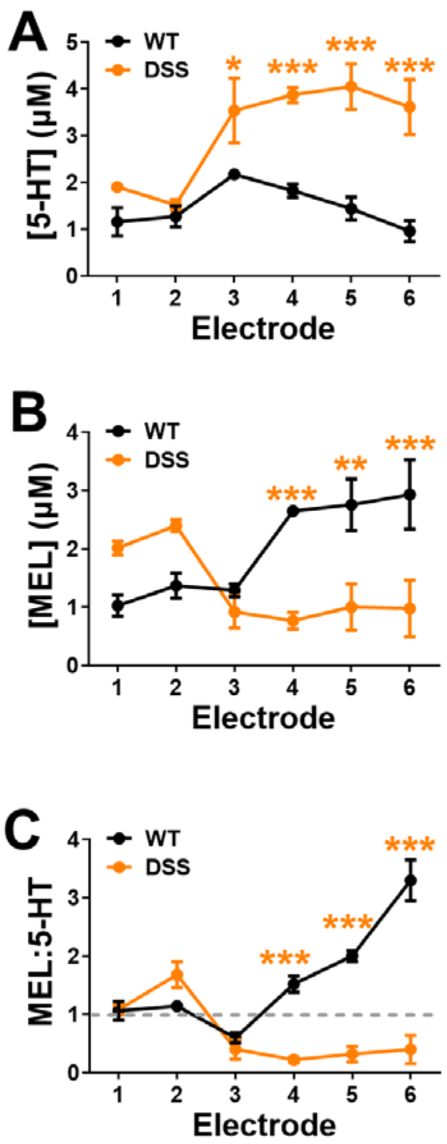

Figure S4. The effect of DSS induced inflammation on mucosal epithelium 5-HT and MEL overflow. Electrode 1 represent the proximal most region of the colon and electrode 6 represent the distal most region of the colon. Changes in (A) 5-HT (B) MEL and (C) MEL:5-HT ratio are shown in WT and DSS-inflamed colons. Data shown as Mean ± St.Dev., n=5, where *P<0.05, **P<0.01 and ***P<0.001.
